# Hospital Meetings

**Published:** 1901-03-02

**Authors:** 


### HOSPITAL MEETINGS.

### CHARING CROSS HOSPITAL.

The Eightieth Annual General Court of Governors of Charing Cross Hospital was held on Wednesday, 20th instant. Mr. Geo. J. Drummond, the treasurer moved a resolution of regret at the death of the Duke of Saxe-Coburg, the President, and one appointing Lord Wantage, V.C., to the vacant office. These were agreed to, and Lord Wantage thereupon took the chair. He moved the following address to the King:—

We, the President, Governors, and Medical Staff of the Charing Cross Hospital assembled at the Annual General Court, desire to convey to your Majesty our profound sorrow at the death of Her Majesty Queen Victoria, for 67 years the Patron and generous supporter of this Hospital, upon which, on 16th December, 1833, the honour of her patronage was conferred; and to offer to your Majesty, to Her Majesty the Queen, and the Royal Family our respectful sympathy.

We, your dutiful subjects, further beg leave humbly to present to your Majesty our loyal congratulations upon your Majesty's Accession to the Throne, and earnestly pray that the Almighty will vouchsafe to your Majesty a prosperous and happy reign, and that His blessing may ever attend your Majesty, our Gracious Queen Alexandra and your Royal House.

This address was adopted, all upstanding.

The annual report of the Council and the accounts for the year ended December 31st, 1900, were then presented. The Council referred with sorrow to the great loss which had fallen on the Empire in the death of Queen Victoria. As mentioned in the Address to the Throne, Her Majesty was patron of the hospital for more than sixty-seven years, and the Victoria Ward was the first of its kind to be so named. The following is the Royal letter of consent received by the hon. secretary of that day, Mr. John Robertson:—

Kensington Palace,
December 16th, 1833.

Sir,—I have submitted to the Duchess of Kent the request of the Governors that the Charing Cross Hospital have the patronage of the Princess Victoria, and that the principal ward for female patients be called "The Victoria Ward," and I have Her Royal Highness's commond to request you will be pleased to inform the Governors that she is most happy to accede to their wishes and identify the Princess's name thus early with the cause of charity and humanity.—I have, &c., John Conroy.

Up to the close of her reign Her Majesty's interest in the hospital was unabated. In 1897 she presented two portraits of herself to the Victoria Ward, in one of which she is represented at the age of fourteen, when she conferred her name on the ward standing by the side of her mother the Duchess of Kent. She gave her patronage to the Albert Hall Bazaar in 1899, and last year her private secretary wrote: "Her Majesty is gratified and interested to hear of the success that is anticipated as regards the enlargement of so excellent and long established an institution."

Regret was also expressed at the death of the late President and gratification at the acceptance of the office by Lord Wantage, who had been a vice-President and most generous supporter since 1883.

The number of new patients treated during the year amounted to 21,416. There were 2,248 in-patients and 19,168 out-patients, including 9,825 cases of accident and emergency. The number of attendances of out-patients during the year was 44,973. The receipts for the year were: For the General Fund (including legacies), £14,652; for the Special Appeal, £3,006; for the Convalescent Home, £1,469—together, £19,127. The annual subscriptions show a slight falling-off, being £2,339, as against £2,360 last year. The Drapers' Company withdrew their annual subscription of £31 10s., in common with their subscriptions to other hospitals, preferring to give a lump sum to the Prince of Wales's Hospital Fund for London. The total maintenance and administration expenditure amounted to £14,989, and deducting £1,916 as the estimated cost of the out-patients and casualties, the cost per bed worked out at £74 13s., and at £5 16s. per in-patient. The slight increase shown was mainly due to the increased cost of coal and gas. In connection with the Special Appeal an exhibit was arranged at the Earl's Court Exhibition as a model hospital, and resulted in a profit of £1,392. A floral ball at the Kensington Palace Hotel, promoted by the Ladies' Committee, produced £370. On the recommendation of the Medical Committee, the Council had opened for out-patients a department for mental disorders, and Dr. Percy Smith had been appointed physician in charge. Mr. E. C. Montgomery, the resident medical officer, had resigned, and Dr. H. S. Clogg, also an old student and formerly a house physician and house surgeon of the hospital, had been appointed in his place.

The hospital was able to respond to the demand of the War Office for medical officers. Mr. H. A. Fairbank, Mr. B. A. Nicholl, Mr. W. Woolliscroft, and Mr. J. H. Whitehead, all of whom had belonged to the resident staff, were appointed civil surgeons, together with Mr. R. W. Collum, an old student. Mr. Charles Gibbs, the junior assistant surgeon joined the Langman Hospital and was made surgeon-in-charge.

The Council expressed regret that little progress had been made during the past year with the improvements, but the delay had been due to unavoidable causes. The new kitchen and the new sanitary annexe for the south wing were approaching completion. The new engineers' workshop was finished, and a new instalment of hot-water and heating apparatus connected with the new boilers. The Council had decided to carry out the work of reconstruction and improvement in two parts. The first contract would include the new nurses' quarters in Chandos Street, new wards and out-patient and casualty departments in King William Street, and on the site of Toole's Theatre. This would entail a temporary reduction of 25 beds, but the work of the hospital would in no way be interfered with or the patients in the wards subjected to discomfort. All the houses in Chandos Street required for the purpose had been pulled down, and as soon as tenders for erecting the new buildings had been accepted, the three houses remaining in King William Street would also be demolished. The main wall of the new building in King William Street was finished, and a similar wall in Chandos Street had been commenced. The working plans which had been the subject of some discussion with the Commissioners of Woods and Forests had been virtually agreed upon and would, doubtless be approved early in the year. One of the conditions on which the Commissioners sold the freehold of the site for the new buildings was that the plans should be approved by them.

During the year 338 patients were received at the Convalescent Home, which is not restricted to patients from the hospital. The Hospital Sunday Fund Council had, now that it had been in existence for over three years, included the home in their list of grants.

The report was adopted, as were votes of thanks to the Finance Committee, the Building Committee, the Medical Committee, the Convalescent Home Committee, the Nursing Committee, the medical and surgical officers, and the honorary solicitors.

A vote of thanks to the Chairman closed the proceedings.

### GREAT NORTHERN CENTRAL HOSPITAL.

The annual general meeting of the Great Northern Central Hospital was held on February 22nd. Sir John Dickson-Poynder, M.P., Chairman of the Committee of Management, presiding.

In moving the adoption of the report the Chairman referred in suitable terms to the death of the Queen and to the fact that the committee had forwarded to the Duke of Cornwall and York, the President of the Hospital, a letter expressing sorrow at the loss sustained by the Royal Family and the Empire, and an assurance of continued loyalty and desire to carry on the work of the institution in a manner that should commend itself to His Royal Highness and to His Majesty the King. He then reviewed the various matters mentioned in the report, which stated that the work showed a further increase, the number of in-patients having been 2,034 against 1,923 in 1899. The number of beds occupied had risen to 143—13 more than last year—and the average stay of each patient was 25·7 days compared with 24·8 in 1899 and 23·1 in 1898. The out-patients numbered 31,361 against 31,189. Although the work continued to increase, the committee had to report a serious falling off in income, the total receipts being £10,988 as against £13,351 in 1899. The contribution of the Ladies' Association, which in 1899 was £2,150, had fallen to £216; while legacies were £2,210 as compared with £3,651. Annual subscriptions, however, showed an increase of £146. The grants of the Saturday and Sunday Funds were both slightly larger—just over £6 in each instance. In addition to the annual subscription of £750 the Prince of Wales's Fund had made a grant of £500, £250 of which was to go towards the cost of providing a laundry. The receipt of £200 was recorded as the result of a matinée given at the Holloway Empire Theatre of Varieties, and £500 from the trustees of Smith's Charity. The ordinary expenditure amounted to £12,688, and the extraordinary, including £128 for the Charter of Incorporation, to £360, the deficiency shown being £2,060. Expenses of maintenance showed an increase, not only due to the extra cost for six months of the ward opened in July 1899, but also to the general rise on the cost of stores, fuel alone showing an increase of £323. Expenses of management were less by £164, but those under the heading of Finance were increased, chiefly by interest on the loan from the bankers, which amounted to £295. After deducting extraordinary expenditure, and the cost of out-patients at 1s. 9d. per head, the expense per bed worked out at £69 10s. 9d.—a few shillings higher that in 1899, but still comparing favourably with that of other London general hospitals. The committee reported with regret the resignation through ill health of Mr. James Cooper, the casualty officer, who for upwards of nine years had rendered valuable services. Mr. Walter Reynolds had resigned his seat on the committee owing to change of residence. The Chairman emphasised the need for additional support to relieve them from the anxiety of an increasing debt, which now amounted to £8,000. He thought, however, they would be stimulated to increased exertions rather than discouraged by the position. He considered that they did not get the support they ought from the borough of Islington. Only one-third of their income came from the neighbourhood, although 70 per cent. of their patients lived there. He thought also something more might be done by appeals in the various places of worship.

The report was adopted, the retiring members of the committee were re-elected, and votes of thanks accorded to the management, the medical staff, and the Ladies' Association. In acknowledging the last named, Mrs. Harkness (the honorary secretary) mentioned that their president (the Baroness Burdett-Coutts) still showed the greatest interest in the work, and took the chair at the meeting in November.

A vote of thanks to the Chairman, warmly congratulating him on his safe return from South Africa, was enthusiastically received, after which the proceedings terminated.

### QUEEN CHARLOTTE'S HOSPITAL.

The annual meeting of Queen Charlotte's Hospital took place at the Hospital on Monday the 25th ult., the Earl of Hardwicke presiding.

In moving the adoption of the annual report and balance sheet, Lord Hardwicke referred to the irreparable loss sustained by the hospital in the death of H.M. Queen Victoria, who had been Patron for fifty-one years. Her reign had been a unique and glorious one; and all connected with this hospital looked back upon those fifty-one years with gratitude for the touching and womanly interest always shown by her; they were proud to have had her for so long as Patron. A paragraph in the report referred to a message received from her only a few days before her death; it was as follows:

"Sir Fleetwood Edwards is desired to say that the Queen was gratified to hear of the special facilities that have been considerately arranged by the Committee in favour of the wives of soldiers and sailors and reservists whose husbands are out in South Africa." It was a special matter for congratulation that the Committee and all concerned had been able to share the national burden of the war in this way; and he believed and hoped that the gallant men would have no cause to complain of the care taken of their wives in the hospital. If good work depended on the number of patients, he thought the hospital could claim to have done good work during the year; the number admitted (1,183) was the largest on record.

The gift of the Lord Mayor (a grant of £225 from his Discretionary Fund) on hearing of the arrangements made for the benefit of the soldiers' wives was as acceptable as it was unexpected. Now that London had so many mayors, he was not aware whether they would follow this example; if they did, their gifts would be very gratefully received. The committee also acknowledged with grateful thanks the grant of £300 to the hospital and £50 to the convalescent home from the Prince of Wales's Hospital Fund, and of £335 8s. 4d. from the Metropolitan Hospital Sunday Fund, and £91 from the Hospital Saturday Fund, as well as of other contributions.

It was always a pleasing duty to call attention to the devoted services of the medical staff; he heard sometimes of friction existing in other hospitals between the medical staff and the management committee; but he could vouch for the perfect understanding that existed at Queen Charlotte's Hospital during the time he had had the honour of being chairman. Both desired the well-being of the hospital, and would always, he trusted, be ready to give or take suggestions.

By the death of the Bishop of London, every charitable institution, and indeed every Christian work throughout London had lost a true friend. Among many splendid characteristics he had the rare gift of a strong personal interest; it seemed sometimes as if he almost possessed the impossible gift of being in two places at once. Among other losses sustained by the hospital were the death of the Rev. George Colin Campbell, after nearly twenty years' chaplaincy, and that of Dr. Grigg, whose efforts to further the interests of the charity they would always remember with gratitude. Dr. Grigg went to South Africa as a civil surgeon, and contracted fever, to which he succumbed.

He did not think they could look forward to any reduction in expenditure. The improvements which had been carried out were planned with a view to economy, but also with a view to ensuring the greatest possible efficiency, and comfort for the patients. He would suggest that persons interested in the patients, as well as writing letters of thanks for benefits received, should show their appreciation of the treatment by becoming subscribers. Anyone reading the report would see that the limits to which it was right to go in expenditure had not been exceeded. Annual subscriptions, except for small endowments, were the only source of income. This year there had been very many claims on everyone, owing to the war in South Africa, the Indian famine, &c.; but he looked forward to the war being shortly over, when the generous public would be able again to give to the old charities. He thanked his colleagues for their uniform kindness and assistance, and formally moved the adoption of the report and accounts.

Major McMahon having seconded the motion, it was carried unanimously.

A list of governors, annual subscribers, and life donors having been passed, the Committee of Management was re-elected; Mr. F. W. Hunt was re-elected hon. architect; Messrs. Dix, Lewis, Cæsar and Co. were re-appointed as auditors; and the usual votes of thanks ended the proceedings.

Those present were:—The Earl of Hardwicke (chairman), Mr. H. A. Harben (vice-chairman), Mr. R. Martin, Major H. J. Royds, Captain Norcliffe Gilpin, Mr. H. Davies, Mr. F. W. Hunt, Mr. Cavendish Bentinck, Mr. A. Stabb, and the Rev. H. W. Casserly.

### TOTTENHAM HOSPITAL.

Mr. C. F. Cory-Wright presided at the annual general meeting of the Tottenham Hospital on February 26th. He congratulated the subscribers on H.R.H. Princess Louise having consented to become president of the hospital, and that they now had a list of patrons, among them being Lord Jersey, the Bishop of Ripon, Lord George Hamilton, Sir Wm. Broadbent, Sir Wm. MacCormac, Mr. Treves, and others. The amount of work accomplished had largely increased, the in-patients numbering 628 as compared with 530. The out-patients numbered 11,634 with 35,532 attendances, as compared with 9,687 and 24,507 attendances. The building was now practically complete, and when the ward they were then occupying was furnished and opened they would have at their disposal 73 beds. All this, of course meant that a great deal more money was required, and unless they could largely increase their subscriptions and donations, or ran into debt, they must close one of their wards. Their income was £3,812, and showed a falling off of £1,500. But in making the comparison they must remember that of this £500 in 1899 was from the Leathersellers for a specific object, and £500 from the Prince of Wales's Fund, so that was £1,000 accounted for, the remaining £500 being from a gradual falling off in subscriptions and donations—not, however, from the poorest quarters, the penny collections, for instance, being £12 more than last year. But all over the country hospitals and charities were complaining that their incomes had fallen off owing to the war in South Africa. It was of course right to support those who were fighting for us, but if the subscriptions to war funds were taken from the hospitals at home he failed to see where the patriotism came in. Their expenditure was £4,262, or about £1,000 more than in the previous year; but not only was there a great increase in the cost of provisions, but they had had an average of 10 in-patients a day more—39 against 29—while there had been 10,000 more attendances in the out-patient department. They had at least £600 to make up, and if they were to keep open the 73 beds they must have more for current maintenance. They were expecting the money from the Jubilee Fund, and if paid that would liquidate the debt and help them in the coming year.

The report was adopted, and Messrs. J. P. Beavan, F. Jenkins, C. T. Maud, and the Rev. F. B. Johnston having been re-elected to the Managing Committee, and Messrs. Richardson, Douston, and Newton having been elected to fill vacancies, the proceedings terminated with a vote of thanks to the Chairman.

